# (*E*)-Ethyl 2-cyano-3-[4-(4,5-diphenyl-1*H*-imidazol-2-yl)phen­yl]acrylate dihydrate

**DOI:** 10.1107/S1600536811006039

**Published:** 2011-02-26

**Authors:** Wei-Xing Liao, Yi-Wen Peng, Li Yu, Xiao-Qing Ning, He-Ping Zeng

**Affiliations:** a School of Chemistry and Environment, South China Normal University, Guangzhou 510006, People’s Republic of China

## Abstract

In the title compound, C_27_H_21_N_3_O_2_·2H_2_O, the three benzene rings attached to the heterocyclic imidazole ring are not coplanar with the latter, making dihedral angles of 14.8 (2), 31.4 (2), and 37.5 (2)°, respectively, for the benzene ring planes in the 2-, 4- and 5-positions. In the crystal, there are two water mol­ecules which serve as connectors between the acrylate mol­ecules and stabilize the structure *via* N—H⋯O, O—H⋯N, C—H⋯O and O—H⋯O hydrogen bonding.

## Related literature

For background to the electronic and photophysical properties of 2,4,5-triaryl­imidazoles, see: Valiyev *et al.* (2007 ▶). For the synthetic procedure, see: Liu *et al.* (2006 ▶). For related structures, see: Fridman *et al.* (2009 ▶).
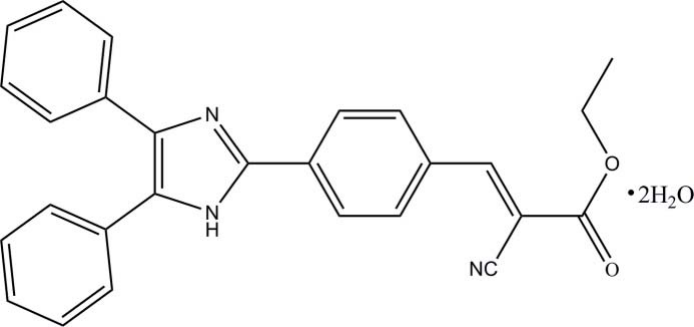


## Experimental

### 

#### Crystal data

C_27_H_21_N_3_O_2_·2H_2_O
*M* *_r_* = 455.50
Triclinic, 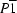

*a* = 8.4976 (16) Å
*b* = 9.0263 (17) Å
*c* = 16.421 (3) Å
α = 83.536 (2)°
β = 79.821 (2)°
γ = 71.089 (2)°
*V* = 1170.7 (4) Å^3^
*Z* = 2
Mo *K*α radiation
μ = 0.09 mm^−1^
*T* = 296 K
0.30 × 0.28 × 0.24 mm

#### Data collection

Bruker APEXII CCD diffractometer
Absorption correction: multi-scan (*SADABS*; Sheldrick, 2004 ▶) *T*_min_ = 0.962, *T*_max_ = 0.969
5922 measured reflections
4048 independent reflections
3010 reflections with *I* > 2σ(*I*)
*R*_int_ = 0.024

#### Refinement

*R*[*F* ^2^ > 2σ(*F* ^2^)] = 0.048
*wR*(*F* ^2^) = 0.134
*S* = 1.03
4048 reflections
326 parameters
9 restraints
H atoms treated by a mixture of independent and constrained refinement
Δρ_max_ = 0.31 e Å^−3^
Δρ_min_ = −0.28 e Å^−3^

### 

Data collection: *APEX2* (Bruker, 2004 ▶); cell refinement: *SAINT* (Bruker, 2004 ▶); data reduction: *SAINT*; program(s) used to solve structure: *SHELXS97* (Sheldrick, 2008 ▶); program(s) used to refine structure: *SHELXL97* (Sheldrick, 2008 ▶); molecular graphics: *SHELXTL* (Sheldrick, 2008 ▶); software used to prepare material for publication: *SHELXL97*.

## Supplementary Material

Crystal structure: contains datablocks global, I. DOI: 10.1107/S1600536811006039/pv2361sup1.cif

Structure factors: contains datablocks I. DOI: 10.1107/S1600536811006039/pv2361Isup2.hkl

Additional supplementary materials:  crystallographic information; 3D view; checkCIF report

## Figures and Tables

**Table 1:** Hydrogen-bond geometry (Å, °)

*D*—H⋯*A*	*D*—H	H⋯*A*	*D*⋯*A*	*D*—H⋯*A*
N1—H1⋯O1*W*	0.88 (3)	2.04 (3)	2.915 (3)	172 (2)
O2*W*—H3*W*⋯N2	0.89 (4)	1.98 (4)	2.870 (3)	173 (3)
C9—H9⋯O1*W*	0.93	2.48	3.338 (3)	154
O1*W*—H2*W*⋯O2*W*^i^	0.86 (4)	1.95 (2)	2.789 (3)	165 (4)
O1*W*—H1*W*⋯N3^ii^	0.83 (2)	2.24 (2)	3.034 (3)	159 (3)
O2*W*—H4*W*⋯O1*W*^iii^	0.81 (4)	2.25 (4)	3.041 (4)	167 (4)

Symmetry codes: (i) 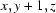; (ii) 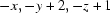; (iii) 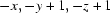.

**Fig. 1.**
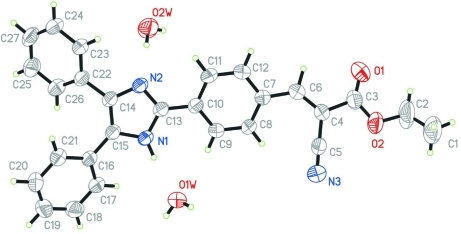
The molecular structure of the title compound, showing the atom-labelling scheme and 50% probability Displacement ellipsoids.

**Fig. 2.**
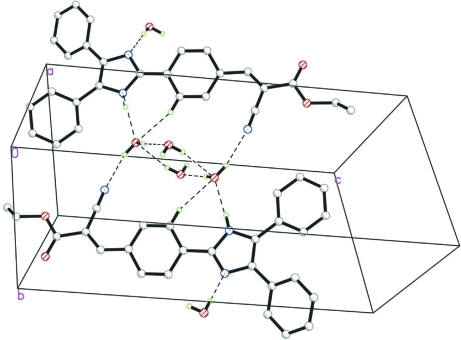
Part view of the crystal structure, showing hydrogen bonds indicated by dashed lines. Hydrogen atoms not involved in H-bonds have been omitted for clarity.

## supplementary crystallographic information

### Comment

2,4,5-Triarylimidazoles based on extended organic π systems have received increasing interest due to the intriguing electronic and photophysical properties (Valiyev *et al.*, 2007). As part of our on-going research interest in chemiluminescence compounds, the title compound was synthesized and its crystal structure determined as described herein.

The molecular structure of the title compound is presented in Fig. 1. Three benzene rings attached to the heterocyclic imidazole ring are not coplanar with the latter, with dihedral angles of 14.8 (2)°, 31.4 (2)°, and 37.5 (2)°, respectively, between the benzene ring planes in the 2-, 4- and 5-positions of the imidazole ring.

In the crystal packing, H_2_O molecules serve as connectors to form the three-dimensional packing *via* hydrogen bonds (Fig. 2, Tab. 1), including N—H···O, O—H···N, C—H···O and O—H···O hydrogen bonds.

The crystal structures of several compounds related to the title molecule have been reported (Fridman *et al.*, 2009).

### Experimental

The title compound was prepared as reported earlier (Liu *et al.*, 2006). A mixture of 4-(4,5-diphenyl-1*H*-imidazol-2-yl)benzaldehyde (0.225 g, 0.69 mmol), ethyl 2-cyanoacetate (0.156 g, 2.1 mmol) and pyridine (2 ml) was stirred at room temperature for 10 h. The solution was poured into water (15 ml) and orange precipitate of the title compound formed immediately. The precipitate obtained was filtered, washed with water and dried. Single crystals of the title compound were obtained by slow evaporation from ethyl acetate at room temperature.

### Refinement

The H-atoms bonded to N and water molecules were located from a difference map and were included at restrained distances O—H = 0.82 (2) and N—H = 0.86 (2) Å. The rest of the H atoms were positioned in calculated positions with C—H = 0.93, 0.96 and 0.97 Å for aryl, methyl and methylene type H-atoms and were refined using a riding model, with *U*_iso_(H) = 1.5 *U*_eq_(C) for methyl H atoms and *U*_iso_ = 1.2*U*_eq_ for others.

### Crystal data

**Table:**

C_27_H_21_N_3_O_2_·2H_2_O	*Z* = 2
*M**_r_* = 455.50	*F*(000) = 480
Triclinic, *P*1	*D*_x_ = 1.292 Mg m^−^^3^
Hall symbol: -P 1	Mo *K*α radiation, λ = 0.71073 Å
*a* = 8.4976 (16) Å	Cell parameters from 2029 reflections
*b* = 9.0263 (17) Å	θ = 2.4–24.2°
*c* = 16.421 (3) Å	µ = 0.09 mm^−^^1^
α = 83.536 (2)°	*T* = 296 K
β = 79.821 (2)°	Block, red
γ = 71.089 (2)°	0.30 × 0.28 × 0.24 mm
*V* = 1170.7 (4) Å^3^	

### Data collection

**Table:**

Bruker APEXII CCD diffractometer	4048 independent reflections
Radiation source: fine-focus sealed tube	3010 reflections with *I* > 2σ(*I*)
graphite	*R*_int_ = 0.024
φ and ω scans	θ_max_ = 25.0°, θ_min_ = 2.4°
Absorption correction: multi-scan (*SADABS*; Sheldrick, 2004)	*h* = −10→10
*T*_min_ = 0.962, *T*_max_ = 0.969	*k* = −10→5
5922 measured reflections	*l* = −18→19

### Refinement

**Table:**

Refinement on *F*^2^	Secondary atom site location: difference Fourier map
Least-squares matrix: full	Hydrogen site location: inferred from neighbouring sites
*R*[*F*^2^ > 2σ(*F*^2^)] = 0.048	H atoms treated by a mixture of independent and constrained refinement
*wR*(*F*^2^) = 0.134	*w* = 1/[σ^2^(*F*_o_^2^) + (0.057*P*)^2^ + 0.3479*P*] where *P* = (*F*_o_^2^ + 2*F*_c_^2^)/3
*S* = 1.03	(Δ/σ)_max_ < 0.001
4048 reflections	Δρ_max_ = 0.31 e Å^−^^3^
326 parameters	Δρ_min_ = −0.28 e Å^−^^3^
9 restraints	Extinction correction: *SHELXL97* (Sheldrick, 2008), Fc^*^=kFc[1+0.001xFc^2^λ^3^/sin(2θ)]^-1/4^
Primary atom site location: structure-invariant direct methods	Extinction coefficient: 0.049 (4)

### Special details

**Table:**

Geometry. All e.s.d.'s (except the e.s.d. in the dihedral angle between two l.s. planes) are estimated using the full covariance matrix. The cell e.s.d.'s are taken into account individually in the estimation of e.s.d.'s in distances, angles and torsion angles; correlations between e.s.d.'s in cell parameters are only used when they are defined by crystal symmetry. An approximate (isotropic) treatment of cell e.s.d.'s is used for estimating e.s.d.'s involving l.s. planes.
Refinement. Refinement of *F*^2^ against ALL reflections. The weighted *R*-factor *wR* and goodness of fit *S* are based on *F*^2^, conventional *R*-factors *R* are based on *F*, with *F* set to zero for negative *F*^2^. The threshold expression of *F*^2^ > σ(*F*^2^) is used only for calculating *R*-factors(gt) *etc*. and is not relevant to the choice of reflections for refinement. *R*-factors based on *F*^2^ are statistically about twice as large as those based on *F*, and *R*- factors based on ALL data will be even larger.

### Fractional atomic coordinates and isotropic or equivalent isotropic displacement parameters (Å^2^)

**Table:**

	*x*	*y*	*z*	*U*_iso_*/*U*_eq_	
N1	0.1176 (2)	0.5581 (2)	0.34629 (10)	0.0461 (4)	
N2	0.2308 (2)	0.30220 (19)	0.36163 (10)	0.0458 (4)	
C13	0.2002 (3)	0.4368 (2)	0.39524 (12)	0.0438 (5)	
C10	0.2495 (2)	0.4536 (2)	0.47340 (12)	0.0426 (5)	
C22	0.1701 (3)	0.2091 (2)	0.23943 (12)	0.0454 (5)	
C4	0.4029 (3)	0.6126 (3)	0.74019 (13)	0.0493 (5)	
C9	0.1825 (3)	0.5922 (2)	0.51442 (13)	0.0476 (5)	
H9	0.1019	0.6761	0.4923	0.057*	
C8	0.2336 (3)	0.6071 (2)	0.58706 (13)	0.0499 (5)	
H8	0.1882	0.7013	0.6131	0.060*	
C14	0.1657 (3)	0.3376 (2)	0.28845 (12)	0.0445 (5)	
C7	0.3527 (3)	0.4828 (2)	0.62223 (12)	0.0458 (5)	
O2	0.4180 (3)	0.7169 (2)	0.86022 (11)	0.0843 (6)	
C12	0.4155 (3)	0.3427 (2)	0.58202 (14)	0.0535 (6)	
H12	0.4921	0.2570	0.6055	0.064*	
C5	0.3509 (3)	0.7711 (3)	0.70713 (14)	0.0532 (6)	
C11	0.3671 (3)	0.3284 (2)	0.50880 (14)	0.0528 (6)	
H11	0.4131	0.2345	0.4825	0.063*	
C15	0.0948 (3)	0.4979 (2)	0.27737 (12)	0.0448 (5)	
C16	0.0128 (3)	0.6002 (2)	0.21090 (13)	0.0473 (5)	
C23	0.3001 (3)	0.0680 (2)	0.24136 (13)	0.0533 (6)	
H23	0.3865	0.0572	0.2715	0.064*	
C26	0.0437 (3)	0.2198 (3)	0.19410 (15)	0.0570 (6)	
H26	−0.0445	0.3130	0.1919	0.068*	
C17	−0.1236 (3)	0.7339 (2)	0.22837 (14)	0.0524 (6)	
H17	−0.1645	0.7595	0.2831	0.063*	
N3	0.3101 (3)	0.8982 (3)	0.68135 (14)	0.0735 (6)	
C3	0.4488 (3)	0.5846 (3)	0.82517 (16)	0.0648 (7)	
C21	0.0700 (3)	0.5658 (3)	0.12820 (14)	0.0595 (6)	
H21	0.1614	0.4773	0.1152	0.071*	
C20	−0.0064 (4)	0.6604 (3)	0.06561 (15)	0.0697 (7)	
H20	0.0333	0.6353	0.0108	0.084*	
C27	0.1752 (3)	−0.0438 (3)	0.15516 (15)	0.0655 (7)	
H27	0.1765	−0.1288	0.1275	0.079*	
C18	−0.1989 (3)	0.8292 (3)	0.16464 (16)	0.0651 (7)	
H18	−0.2891	0.9191	0.1768	0.078*	
C25	0.0461 (3)	0.0946 (3)	0.15209 (16)	0.0645 (7)	
H25	−0.0397	0.1041	0.1218	0.077*	
C24	0.3018 (3)	−0.0562 (3)	0.19897 (15)	0.0636 (7)	
H24	0.3902	−0.1494	0.2002	0.076*	
O1	0.5041 (3)	0.4573 (3)	0.85780 (13)	0.1021 (8)	
C19	−0.1412 (4)	0.7919 (3)	0.08367 (17)	0.0717 (7)	
H19	−0.1932	0.8554	0.0412	0.086*	
O1W	0.0015 (3)	0.8867 (2)	0.38559 (12)	0.0792 (6)	
O2W	0.2110 (3)	0.0132 (3)	0.44859 (16)	0.0886 (7)	
C1	0.5089 (4)	0.8081 (4)	0.96681 (13)	0.1127 (12)	
H1A	0.4386	0.9135	0.9565	0.169*	
H1B	0.6168	0.7918	0.9330	0.169*	
H1C	0.5229	0.7914	1.0242	0.169*	
C2	0.4361 (4)	0.7061 (4)	0.94811 (13)	0.1250 (14)	
H2A	0.3257	0.7256	0.9813	0.150*	
H2B	0.5032	0.6002	0.9629	0.150*	
H2W	0.071 (4)	0.931 (4)	0.396 (2)	0.144 (17)*	
H1W	−0.065 (4)	0.952 (4)	0.357 (2)	0.128 (14)*	
H3W	0.226 (4)	0.101 (4)	0.423 (2)	0.109 (12)*	
H4W	0.168 (5)	0.031 (5)	0.496 (3)	0.145 (18)*	
H1	0.088 (3)	0.659 (3)	0.3534 (15)	0.071 (8)*	
C6	0.4077 (3)	0.4897 (3)	0.69979 (13)	0.0518 (5)	
H6	0.4542	0.3924	0.7262	0.062*	

### Atomic displacement parameters (Å^2^)

**Table:**

	*U*^11^	*U*^22^	*U*^33^	*U*^12^	*U*^13^	*U*^23^
N1	0.0584 (11)	0.0352 (10)	0.0444 (10)	−0.0135 (8)	−0.0060 (8)	−0.0072 (8)
N2	0.0525 (10)	0.0375 (9)	0.0467 (10)	−0.0127 (8)	−0.0049 (8)	−0.0077 (8)
C13	0.0478 (11)	0.0381 (11)	0.0446 (11)	−0.0136 (9)	−0.0025 (9)	−0.0054 (9)
C10	0.0469 (11)	0.0395 (11)	0.0421 (11)	−0.0152 (9)	−0.0030 (9)	−0.0048 (9)
C22	0.0540 (12)	0.0395 (11)	0.0432 (11)	−0.0165 (10)	−0.0012 (9)	−0.0078 (9)
C4	0.0507 (12)	0.0489 (13)	0.0483 (12)	−0.0128 (10)	−0.0135 (10)	−0.0018 (10)
C9	0.0506 (12)	0.0399 (11)	0.0494 (12)	−0.0075 (9)	−0.0115 (9)	−0.0037 (9)
C8	0.0575 (13)	0.0401 (12)	0.0510 (13)	−0.0090 (10)	−0.0125 (10)	−0.0102 (10)
C14	0.0489 (12)	0.0401 (11)	0.0441 (11)	−0.0139 (9)	−0.0024 (9)	−0.0083 (9)
C7	0.0507 (12)	0.0415 (12)	0.0457 (12)	−0.0146 (10)	−0.0088 (9)	−0.0014 (9)
O2	0.1337 (17)	0.0820 (13)	0.0525 (10)	−0.0434 (12)	−0.0331 (11)	−0.0062 (9)
C12	0.0592 (13)	0.0409 (12)	0.0558 (13)	−0.0066 (10)	−0.0149 (11)	−0.0013 (10)
C5	0.0589 (14)	0.0530 (15)	0.0506 (13)	−0.0149 (11)	−0.0151 (11)	−0.0111 (11)
C11	0.0632 (14)	0.0361 (12)	0.0539 (13)	−0.0069 (10)	−0.0080 (11)	−0.0087 (10)
C15	0.0523 (12)	0.0395 (11)	0.0428 (11)	−0.0147 (9)	−0.0041 (9)	−0.0067 (9)
C16	0.0584 (13)	0.0410 (11)	0.0463 (12)	−0.0192 (10)	−0.0085 (10)	−0.0061 (9)
C23	0.0652 (14)	0.0421 (12)	0.0506 (12)	−0.0120 (11)	−0.0081 (10)	−0.0089 (10)
C26	0.0577 (14)	0.0511 (13)	0.0651 (14)	−0.0188 (11)	−0.0074 (11)	−0.0126 (11)
C17	0.0606 (14)	0.0440 (12)	0.0542 (13)	−0.0170 (11)	−0.0082 (10)	−0.0074 (10)
N3	0.0945 (17)	0.0497 (13)	0.0759 (15)	−0.0122 (12)	−0.0292 (12)	−0.0064 (11)
C3	0.0772 (17)	0.0685 (17)	0.0583 (15)	−0.0290 (14)	−0.0267 (13)	0.0015 (13)
C21	0.0755 (16)	0.0504 (13)	0.0501 (13)	−0.0155 (12)	−0.0066 (11)	−0.0094 (11)
C20	0.103 (2)	0.0661 (17)	0.0465 (14)	−0.0301 (16)	−0.0181 (13)	−0.0061 (12)
C27	0.0919 (19)	0.0530 (15)	0.0589 (15)	−0.0327 (14)	−0.0016 (13)	−0.0186 (12)
C18	0.0670 (15)	0.0528 (14)	0.0757 (17)	−0.0120 (12)	−0.0241 (13)	−0.0028 (12)
C25	0.0711 (16)	0.0658 (16)	0.0677 (16)	−0.0316 (14)	−0.0135 (12)	−0.0136 (13)
C24	0.0846 (18)	0.0405 (13)	0.0599 (14)	−0.0108 (12)	−0.0067 (13)	−0.0123 (11)
O1	0.154 (2)	0.0779 (14)	0.0811 (14)	−0.0278 (14)	−0.0646 (14)	0.0158 (11)
C19	0.0910 (19)	0.0665 (17)	0.0666 (17)	−0.0261 (15)	−0.0377 (15)	0.0044 (13)
O1W	0.1181 (17)	0.0475 (11)	0.0756 (13)	−0.0201 (11)	−0.0391 (12)	0.0042 (9)
O2W	0.136 (2)	0.0639 (13)	0.0736 (15)	−0.0457 (13)	−0.0134 (14)	0.0031 (11)
C1	0.161 (3)	0.132 (3)	0.0659 (19)	−0.063 (3)	−0.031 (2)	−0.017 (2)
C2	0.214 (4)	0.151 (3)	0.0583 (18)	−0.111 (3)	−0.053 (2)	0.0087 (19)
C6	0.0549 (13)	0.0468 (12)	0.0518 (13)	−0.0113 (10)	−0.0141 (10)	0.0015 (10)

### Geometric parameters (Å, °)

**Table:**

N1—C13	1.357 (3)	C23—C24	1.379 (3)
N1—C15	1.378 (3)	C23—H23	0.9300
N1—H1	0.88 (3)	C26—C25	1.381 (3)
N2—C13	1.321 (2)	C26—H26	0.9300
N2—C14	1.373 (3)	C17—C18	1.386 (3)
C13—C10	1.456 (3)	C17—H17	0.9300
C10—C9	1.391 (3)	C3—O1	1.195 (3)
C10—C11	1.393 (3)	C21—C20	1.373 (3)
C22—C26	1.385 (3)	C21—H21	0.9300
C22—C23	1.391 (3)	C20—C19	1.374 (4)
C22—C14	1.471 (3)	C20—H20	0.9300
C4—C6	1.342 (3)	C27—C24	1.366 (4)
C4—C5	1.427 (3)	C27—C25	1.373 (4)
C4—C3	1.487 (3)	C27—H27	0.9300
C9—C8	1.373 (3)	C18—C19	1.373 (4)
C9—H9	0.9300	C18—H18	0.9300
C8—C7	1.393 (3)	C25—H25	0.9300
C8—H8	0.9300	C24—H24	0.9300
C14—C15	1.380 (3)	C19—H19	0.9300
C7—C12	1.394 (3)	O1W—H2W	0.86 (4)
C7—C6	1.446 (3)	O1W—H1W	0.834 (18)
O2—C3	1.314 (3)	O2W—H3W	0.89 (4)
O2—C2	1.46737	O2W—H4W	0.81 (4)
C12—C11	1.369 (3)	C1—C2	1.3504
C12—H12	0.9300	C1—H1A	0.9600
C5—N3	1.140 (3)	C1—H1B	0.9600
C11—H11	0.9300	C1—H1C	0.9600
C15—C16	1.468 (3)	C2—H2A	0.9700
C16—C17	1.392 (3)	C2—H2B	0.9700
C16—C21	1.393 (3)	C6—H6	0.9300
			
C13—N1—C15	108.19 (17)	C25—C26—H26	119.4
C13—N1—H1	128.2 (16)	C22—C26—H26	119.4
C15—N1—H1	123.5 (16)	C18—C17—C16	120.3 (2)
C13—N2—C14	106.65 (16)	C18—C17—H17	119.8
N2—C13—N1	110.47 (18)	C16—C17—H17	119.8
N2—C13—C10	124.99 (18)	O1—C3—O2	124.4 (2)
N1—C13—C10	124.53 (18)	O1—C3—C4	124.1 (2)
C9—C10—C11	118.36 (19)	O2—C3—C4	111.5 (2)
C9—C10—C13	121.92 (18)	C20—C21—C16	121.1 (2)
C11—C10—C13	119.73 (18)	C20—C21—H21	119.5
C26—C22—C23	117.82 (19)	C16—C21—H21	119.5
C26—C22—C14	122.29 (19)	C21—C20—C19	120.3 (2)
C23—C22—C14	119.80 (19)	C21—C20—H20	119.9
C6—C4—C5	123.56 (19)	C19—C20—H20	119.9
C6—C4—C3	118.9 (2)	C24—C27—C25	119.6 (2)
C5—C4—C3	117.5 (2)	C24—C27—H27	120.2
C8—C9—C10	121.08 (19)	C25—C27—H27	120.2
C8—C9—H9	119.5	C19—C18—C17	120.5 (2)
C10—C9—H9	119.5	C19—C18—H18	119.8
C9—C8—C7	120.76 (19)	C17—C18—H18	119.8
C9—C8—H8	119.6	C27—C25—C26	120.0 (2)
C7—C8—H8	119.6	C27—C25—H25	120.0
N2—C14—C15	109.59 (17)	C26—C25—H25	120.0
N2—C14—C22	119.24 (17)	C27—C24—C23	120.8 (2)
C15—C14—C22	131.02 (19)	C27—C24—H24	119.6
C8—C7—C12	117.82 (19)	C23—C24—H24	119.6
C8—C7—C6	123.33 (19)	C18—C19—C20	119.8 (2)
C12—C7—C6	118.79 (19)	C18—C19—H19	120.1
C3—O2—C2	117.12	C20—C19—H19	120.1
C11—C12—C7	121.5 (2)	H2W—O1W—H1W	107 (2)
C11—C12—H12	119.2	H3W—O2W—H4W	109 (4)
C7—C12—H12	119.2	C2—C1—H1A	109.5
N3—C5—C4	179.3 (2)	C2—C1—H1B	109.5
C12—C11—C10	120.42 (19)	H1A—C1—H1B	109.5
C12—C11—H11	119.8	C2—C1—H1C	109.5
C10—C11—H11	119.8	H1A—C1—H1C	109.5
N1—C15—C14	105.09 (18)	H1B—C1—H1C	109.5
N1—C15—C16	121.59 (18)	C1—C2—O2	113.40
C14—C15—C16	133.31 (19)	C1—C2—H2A	108.9
C17—C16—C21	118.1 (2)	O2—C2—H2A	108.9
C17—C16—C15	121.25 (19)	C1—C2—H2B	108.9
C21—C16—C15	120.7 (2)	O2—C2—H2B	108.9
C24—C23—C22	120.5 (2)	H2A—C2—H2B	107.7
C24—C23—H23	119.7	C4—C6—C7	131.0 (2)
C22—C23—H23	119.7	C4—C6—H6	114.5
C25—C26—C22	121.2 (2)	C7—C6—H6	114.5
			
C14—N2—C13—N1	0.0 (2)	N1—C15—C16—C17	−38.2 (3)
C14—N2—C13—C10	−179.44 (19)	C14—C15—C16—C17	143.4 (2)
C15—N1—C13—N2	−0.3 (2)	N1—C15—C16—C21	141.8 (2)
C15—N1—C13—C10	179.10 (18)	C14—C15—C16—C21	−36.7 (4)
N2—C13—C10—C9	−165.5 (2)	C26—C22—C23—C24	0.3 (3)
N1—C13—C10—C9	15.2 (3)	C14—C22—C23—C24	176.9 (2)
N2—C13—C10—C11	14.7 (3)	C23—C22—C26—C25	0.0 (3)
N1—C13—C10—C11	−164.7 (2)	C14—C22—C26—C25	−176.5 (2)
C11—C10—C9—C8	1.5 (3)	C21—C16—C17—C18	−0.1 (3)
C13—C10—C9—C8	−178.4 (2)	C15—C16—C17—C18	179.8 (2)
C10—C9—C8—C7	−0.8 (3)	C2—O2—C3—O1	−6.81
C13—N2—C14—C15	0.4 (2)	C2—O2—C3—C4	171.99
C13—N2—C14—C22	−175.68 (18)	C6—C4—C3—O1	6.8 (4)
C26—C22—C14—N2	145.5 (2)	C5—C4—C3—O1	−175.1 (3)
C23—C22—C14—N2	−30.9 (3)	C6—C4—C3—O2	−172.1 (2)
C26—C22—C14—C15	−29.5 (3)	C5—C4—C3—O2	6.0 (3)
C23—C22—C14—C15	154.0 (2)	C17—C16—C21—C20	−0.4 (4)
C9—C8—C7—C12	−1.1 (3)	C15—C16—C21—C20	179.7 (2)
C9—C8—C7—C6	−178.1 (2)	C16—C21—C20—C19	0.2 (4)
C8—C7—C12—C11	2.3 (3)	C16—C17—C18—C19	0.8 (4)
C6—C7—C12—C11	179.4 (2)	C24—C27—C25—C26	−0.8 (4)
C6—C4—C5—N3	−166 (25)	C22—C26—C25—C27	0.3 (4)
C3—C4—C5—N3	16 (25)	C25—C27—C24—C23	1.1 (4)
C7—C12—C11—C10	−1.6 (3)	C22—C23—C24—C27	−0.8 (4)
C9—C10—C11—C12	−0.3 (3)	C17—C18—C19—C20	−0.9 (4)
C13—C10—C11—C12	179.6 (2)	C21—C20—C19—C18	0.4 (4)
C13—N1—C15—C14	0.5 (2)	C3—O2—C2—C1	139.63
C13—N1—C15—C16	−178.30 (18)	C5—C4—C6—C7	−5.9 (4)
N2—C14—C15—N1	−0.5 (2)	C3—C4—C6—C7	172.0 (2)
C22—C14—C15—N1	174.9 (2)	C8—C7—C6—C4	−21.8 (4)
N2—C14—C15—C16	178.1 (2)	C12—C7—C6—C4	161.2 (2)
C22—C14—C15—C16	−6.5 (4)		

### Hydrogen-bond geometry (Å, °)

**Table:**

*D*—H···*A*	*D*—H	H···*A*	*D*···*A*	*D*—H···*A*
N1—H1···O1W	0.88 (3)	2.04 (3)	2.915 (3)	172 (2)
O2W—H3W···N2	0.89 (4)	1.98 (4)	2.870 (3)	173 (3)
C9—H9···O1W	0.93	2.48	3.338 (3)	154
C6—H6···O1	0.93	2.44	2.813 (3)	104
C2—H2B···O1	0.97	2.27	2.680 (3)	105
O1W—H2W···O2W^i^	0.86 (4)	1.95 (2)	2.789 (3)	165 (4)
O1W—H1W···N3^ii^	0.83 (2)	2.24 (2)	3.034 (3)	159 (3)
O2W—H4W···O1W^iii^	0.81 (4)	2.25 (4)	3.041 (4)	167 (4)

Symmetry codes: (i) *x*, *y*+1, *z*; (ii) −*x*, −*y*+2, −*z*+1; (iii) −*x*, −*y*+1, −*z*+1.
